# Problem solving and collaborative involvement among adolescents with spinal cord injury and their caregivers

**DOI:** 10.3389/fresc.2023.1100707

**Published:** 2023-06-26

**Authors:** Olivia E. Clark, Anne L. Rivelli, Alison L. Mroczkowski, Susan Ryerson Espino, Erin Hayes Kelly, Lawrence C. Vogel, Kathy Zebracki

**Affiliations:** ^1^ Department of Psychology, Loyola University Chicago, Chicago, IL, United States; ^2^ Advocate Aurora Research Institute, Advocate Aurora Health, Downers Grove, IL, United States; ^3^ Museum of Science and Industry, Chicago, IL, United States; ^4^ Ryerson Espino Evaluation and Development Consulting, Arlington Heights, IL, United States; ^5^ American Academy of Pediatrics, Itasca, IL, United States; ^6^ Department of Disability & Human Development, University of Illinois at Chicago, Chicago, IL, United States; ^7^ Shriners Children's Chicago, Chicago, IL, United States; ^8^ Department of Pediatrics, Rush Medical College, Chicago, IL, United States; ^9^ Department of Psychiatry and Behavioral Science, Northwestern University Feinberg School of Medicine, Chicago, IL, United States

**Keywords:** spinal cord injury, pediatric, adolescent, caregiver, problem solving, collaboration, rehabilitation

## Abstract

**Objective:**

To determine the relationship between constructive adolescent problem solving (positive problem-solving orientation and rational problem-solving style) and caregiver problem solving and collaborative involvement with primary caregiver among adolescents with spinal cord injuries (SCIs). Positive constructive adolescent problem solving was hypothesized to be predicted by more effective caregiver problem solving and higher collaborative involvement.

**Methods:**

Participants in this cross-sectional study were 79 adolescent and primary caregiver dyads recruited from a pediatric rehabilitation care system in North America. All participants completed a standardized problem-solving instrument and adolescent participants completed an adapted measure of collaborative parent involvement.

**Results:**

More effective caregiver problem solving and adolescent perceptions of more collaboration with caregivers around SCI care were significantly associated with higher positive problem-solving orientation and higher rational problem-solving style among adolescents.

**Conclusions:**

Results underscore the importance of caregiver problem-solving skills and their collaboration with adolescents with SCI when addressing care needs. Clinically, findings highlight opportunities for parent involvement and skill-building as an important factor of rehabilitation for adolescents with SCI.

## 1. Introduction

Adolescents with spinal cord injuries (SCIs) may face a variety of medical, physical, and psychological challenges owing to their injury. Youth with SCI must navigate functional limitations, motor and sensory impairments, autonomic dysfunction including bowel, bladder, and sexual dysfunction, accessibility issues, and the need for ongoing therapies, superimposed on the typical developmental challenges of adolescence (1). These challenges affect a variety of functional domains across several contexts and may change as the youth ages, necessitating frequent attempts to solve problems related to impairments. For youth with SCI, caregivers play an especially important role in their lives by helping to navigate the unique set of challenges related to their SCI-specific care (2–5). As a result, youth with SCI may rely on caregivers to manage more of their needs, or at the very least look to them for guidance on how to problem-solve the unique needs of their SCI, as compared to adolescents without extra healthcare needs. Understanding contributors to problem-solving abilities in youth with SCI is especially important.

Problem solving is the process of understanding and responding to problems to change a difficult situation and to reduce emotional stress (6). Constructive problem-solving includes a positive orientation and a rational style and is associated with adaptive functioning and positive psychological wellbeing. In contrast, dysfunctional problem-solving is associated with maladaptive functioning and psychological distress and involves negative orientation, impulsivity/carelessness, and avoidant style (7, 8). Parent problem-solving has been shown to be associated with health-related quality of life among children with SCI (7), suggesting that caregiver problem-solving may also impact adolescent self-management of medical needs. Exploring the association between caregiver problem-solving approaches, particularly ones that support constructive problem-solving among adolescents with SCI as they navigate varied challenges, is crucial.

While the association between parent and adolescent problem-solving has not been established among adolescents with SCI, problem solving among youth with other chronic conditions and adults with SCI can inform hypotheses. Among adolescents with traumatic brain injury (TBI), problem-solving interventions have been shown to be associated with improved long-term executive function (10) and reduction in behavioral problems (11). Further, among adults with SCI, positive problem-solving has been associated with adaptive and protective wellness behaviors, increased assertiveness and disability acceptance, and less depression and psychosocial impairment (12–14). In contrast, negative problem-solving has been associated with increased risk behaviors among adults with SCI (12). Caregivers, often parents, play an important role in the lives of adolescents with spinal cord injury or dysfunction (3–5). Given the multidimensionality of pediatric SCI and because there is limited research regarding problem solving among adolescents with SCI, a better understanding of the relationship between constructive problem-solving in adolescents with SCI specifically and their parents' problem-solving approaches, constructive or dysfunctional, as well as adolescents' perceptions of their involvement in care, is necessary.

Development of independence and a sense of responsibility are critical developmental characteristics of adolescence. These characteristics are often achieved through repeated efforts to solve problems. In adolescents with SCI, these problems may include those associated with managing their care, including managing health-related medical regimens and activity adjustments (12). Collaboration between caregivers and adolescents around SCI care, which may support problem-solving in these situations, entails discussing care needs, assessing and developing care strategies together, and anticipating challenges in multiple settings [home, school and peer contexts; (15)]. Indeed, among adolescents with type 1 diabetes who perceived a collaborative approach to care with their parents, a protective role in health care management and health-related quality of life for collaborative parent involvement was identified (16). Youth with SCI, because of the complexity and variety of challenges associated (e.g., medical, mobility, psychosocial), may necessitate further parent involvement to support appropriate care management and activity modification. However, little is known about the role of collaborative care among adolescents with SCI and their caregivers. A collaborative approach to care, the ability of caregivers and adolescents to work together regarding adolescent healthcare in a fashion that supports transition of responsibility to the adolescent, may parallel processes of adolescent problem-solving in their daily lives (16). A supportive and collaborative approach may foster developmentally appropriate independence while ensuring that medical care needs are met.

This cross-sectional study investigates individual and combined associations between caregiver problem-solving approaches and adolescent-reported collaborative involvement in care (predictors) and constructive problem-solving in adolescents with SCI (outcome). More effective overall caregiver problem solving and higher levels of collaborative parent involvement were hypothesized to each significantly predict positive (Hypothesis 1) and rational (Hypothesis 2) adolescent problem solving.

## 2. Materials and methods

### 2.1. Participants

Adolescents aged 13 to 18 years with a traumatic SCI of at least one-year duration and their primary caregivers, mostly parents, were included in the present study. Participants were recruited as part of a larger study assessing relationships among psychosocial outcomes in youth with SCI between the ages of one and 18 years and their primary caregivers (Table 1). Of the 150 caregiver-child dyads who participated in the larger study, 71 dyads were excluded as the child was younger than 13 years of age. Adolescent participants received care at one of three specialty hospitals in the same healthcare system, located across the United States. Adolescents and caregivers were excluded if they had significant cognitive impairment as identified by their treating physician, psychologist, social worker, or medical chart review. Institutional Review Board approval was obtained and ethical treatment of human subjects was followed throughout the research process. Caregivers and adolescents who were at least 18 years old provided consent, and adolescents who were 17 years old or younger provided assent. This was an observational study using a convenience sample. There was only one target group (adolescents with SCI and their caregiver); therefore, the sample was not randomized, and researchers were not blinded. Moreover, a power analysis was not conducted.

**Table 1:** Demographic and injury characteristics of adolescent and caregiver participants.

	Adolescent participants (*n *= 79)	Primary caregivers (*n *= 79)
Sex		
Male	44 (56%)	7 (13%)
Female	35 (44%)	48 (87%)
Race		
Caucasian	52 (66%)	52 (66%)
Hispanic	15 (19%)	17 (22%)
African American	3 (4%)	3 (4%)
Asian	2 (3%)	3 (4%)
Native American	2 (3%)	2 (3%)
Other	2 (3%)	0 (0%)
Not reported	3 (4%)	2 (3%)
Mean age at time of interview	15.8 years (*SD *= 1.6)	45.1 years (*SD *= 6.5)
Mean age at time of injury	8.7 years (*SD *= 5.6)	–
Level of injury		
Paraplegia	52 (66%)	–
Tetraplegia	27 (34%)	–
Neurological Category		
AIS A*	34 (44%)	–
AIS B, C, D*	32 (41%)	–
Missing	12 (15%)	–
Cause of injury		
Motor vehicle/pedestrian injuries	38 (48%)	–
Medical/surgical	12 (15%)	–
Sporting activities	10 (13%)	–
Transverse myelitis	9 (11%)	–
Gunshots	6 (8%)	–
Falls/falling objects	4 (5%)	–
Primary caregiver's relationship to adolescent		
Mother	–	66 (84%)
Father	–	8 (10%)
Grandmother	–	2 (3%)
Other	–	3 (4%)
Primary caregiver's education		
Less than a high school degree	–	10 (13%)
High school graduate	–	32 (41%)
Associates degree or higher		37 (47%)
Primary caregiver's marital status		
Married	–	42 (53%)
Not married	–	32 (41%)
Not reported	–	5 (6%)
Family's country of primary residence		
United States	67 (85%)	
Mexico	7 (9%)	
Canada	4 (5%)	
Other	1 (1%)	

* AIS, American spinal injury association impairment scale.

Participants were recruited during inpatient or outpatient visits. Seventy-nine adolescent-caregiver participant dyads completed measures of problem-solving (adolescent and caregiver) and collaborative parent involvement (adolescent only). Research assistants were available if participants requested help completing the measures, which were available in both paper on online versions. Participants completed surveys at home if time did not allow during their visit. Total survey administration typically took 45–75 min. The surveys examined in the current study were completed in approximately 20 min.

### 2.2. Measures

#### 2.2.1. Demographic information

Adolescents' age, sex, and injury-related information were obtained from their medical records. Caregiver sex, relationship to adolescent, marital status, and educational attainment were gathered from a project specific questionnaire.

#### 2.2.2. Problem solving

*The Social Problem-Solving Inventory*, revised, short form (SPSI-R:S) was completed by both adolescents and caregivers. The SPSI-R:S is a 25-item self-report measure yielding five subscale scores, including two assessing problem-solving orientation (Positive and Negative) and three assessing problem-solving style (Rational, Impulsivity/Carelessness, and Avoidance (6);. Positive problem-solving orientation (PPO) and rational problem-solving style (RPS) are conceptualized as constructive problem-solving dimensions, and negative problem-solving orientation (NPO), impulsivity/carelessness style (ICS) and avoidance style (AS) as dysfunctional dimensions. A total problem-solving score is computed by adding the positive orientation and rational style subscale scores to reversed-scored negative orientation, impulsivity/carelessness, and avoidance style subscale scores; this total score allows for an overall indicator of problem-solving effectiveness, with a higher score indicating more effective problem-solving. The SPSI-R:S has been found to be reliable and valid (17). Subscales and total scores demonstrated acceptable internal consistency reliability with the current sample of adolescents and primary caregivers (α = .78-.85), except for caregiver avoidant problem-solving style (α = .62) and adolescent impulsivity/carelessness problem-solving style (α = .59). For the purposes of this study, all subscale scores and the total score were explored among caregivers, and only constructive subscales, specifically PPO and RPS subscales, were explored among adolescents.

#### 2.2.3. Collaborative parent involvement in SCI care (CPI-SCI)

**Figure 1:**
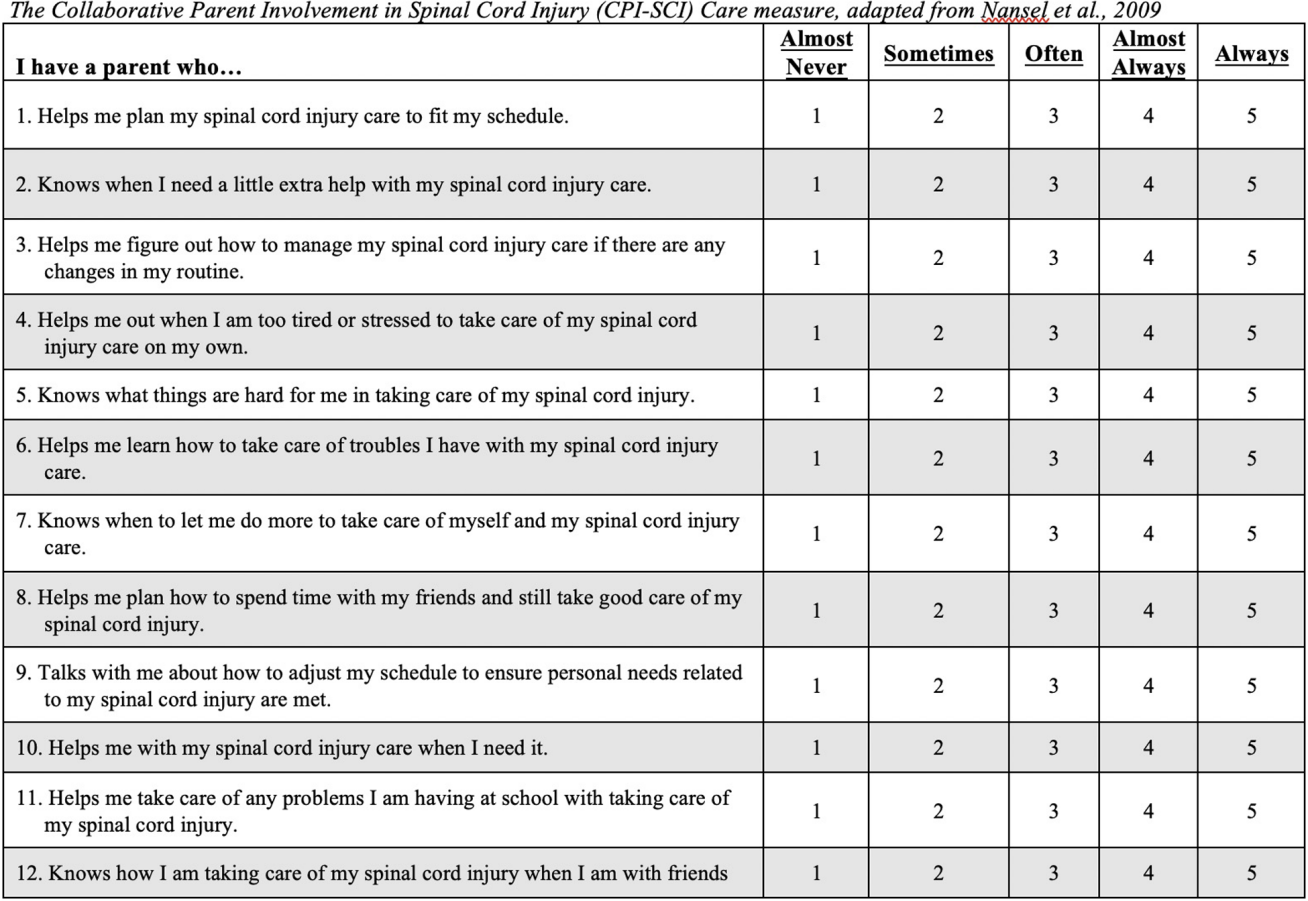
The collaborative Parent Involvement in Spinal Cord Injury (CPI-SCI Care measure, adapted from et al., 2009.

The CPI-SCI was adapted from the *Collaborative Parent Involvement Scale* (CPI (16); and was completed by adolescents. The CPI is a 12-item Likert scale measure developed to assess adolescent perceptions of collaborative parental behavior in diabetes management from the perspective of youth with type I diabetes. The CPI was adapted for the current study by maintaining the intent of each of the CPI items but modified for pediatric SCI (e.g., “diabetes care” was changed to “spinal cord injury care”) and to better match the care needs of youth with SCI (e.g., “…talks with me about how to adjust my insulin, eating, and exercise” was changed to “…talks with me about how to adjust my schedule to ensure personal needs related to my spinal cord injury are met”). See Figure 1 for all adapted items included in the CPI-SCI. When completing the CPI-SCI, adolescents were given the following prompt: “These questions ask about your spinal cord injury care, such as bowel and bladder management, skin care, transfers, and dressing.” For each question, adolescents rated their parent involvement on a scale of 1 (*Almost Never*) to 5 (*Always*). Total scores ranged from 12 to 60 and means were used to assess overall level of collaboration with primary caregiver, with higher scores indicating a greater adolescent-reported level of collaboration between the caregiver and adolescent with SCI. The CPI-SCI demonstrated excellent reliability with the current sample of adolescents (α = .95).

### 2.3. Data analysis

Descriptive statistics examined demographic variables (Table 1). Pearson correlation coefficients were then computed to assess bivariate relationships between subscale and total caregiver SPSI:R-S scores and CPI-SCI scores (predictors), and adolescent positive problem-solving orientation and rational problem-solving style (outcomes; Table 2). Paired sample t-tests examined mean difference scores across parent and adolescent report of positive problem-solving orientation and rational problem-solving styles.

**Table 2:** Correlations among adolescent problem-solving, caregiver problem-solving, and collaborative parent involvement.

	Caregiver–PPO^a^ subscale	Caregiver–NPO^b^ subscale	Caregiver–RPS^c^ subscale	Caregiver–ICS^d^ subscale	Caregiver–AS^e^ subscale	Caregiver–Total Problem-solving Score	Adolescent report –CPI-SCI^+^	Descriptive Statistics
Adolescent–PPO subscale	.34**	−.22	.30**	−.07	−.12	.33**	.27*	*M = *13.2 (*SD *= 4.9) *Range *= 0–20 *α* = .85
Adolescent–RPS subscale	.29*	−.33**	.26*	−.21	−.22*	.40**	.25*	*M *= 10.7 (*SD *= 4.8) *Range *= 0–19 α = .84
Descriptive Statistics	M = 12.5 (SD = 4.3) Range = 0–20 α = .80	M = 4.5 (SD = 3.9) Range = 0–19 α = .81	M = 11.5 (SD = 4.2) Range = 4–20 α = .82	M = 4.5 (SD = 4.1) Range = 0–20 α = .81	M = 3.6 (SD = 3.0) Range = 0–12 α = .62	M = 14.3 (SD = 2.6) Range = 6–19.6 α = .67	M = 4.3 (SD = 0.93) Range = 1–5 α = .95	–

^+^ CPI-SCI was not significantly correlated to any of the caregiver problem-solving variables.

* *p* < .05.

** *p* < .01.

^a^ Positive Problem-solving Orientation (PPO).

^b^ Negative Problem-solving Orientation (NPO).

^c^ Rational Problem-solving Style (RPS).

^d^ Impulsivity/Carelessness Problem-solving Style (ICS).

^e^ Avoidance Problem-solving Style (AS).

Multivariable linear regression models using caregiver problem-solving and adolescent-report of caregiver collaborative involvement as predictors of (1) adolescent positive problem-solving orientation (Table 3) and (2) adolescent rational problem-solving style (Table 4) were then conducted. Crude relationships between adolescent and caregiver problem solving are described, as well as models subsequently adjusted for adolescent-report of caregiver collaborative involvement. The data were analyzed using IBM SPSS Statistics (Version 27; IBM Corp, 2020).

**Table 3:** Multivariable linear regression predicting adolescent positive problem-solving orientation.

	Model							
		*B*	*SE*	*β*	*F*	*R*	*R^2^*	*ΔR^2^*
1	(Constant)	4.44	2.94					
	Caregiver SPSI-R Total Score	.61	.20	.33**	9.09**	.33	.09	.09
2	(Constant)	−1.92	3.72					
	Caregiver SPSI-R Total Score	.62	.20	.33**				
	Adolescent report CPI-SCI	1.45	.55	.27*	8.36**	.43	.16	.07

* *p* < .05.

** *p *< .01.

**Table 4:** Multivariable linear regression predicting adolescent rational problem-solving style.

	Model							
		*B*	*SE*	*β*	*F*	*R*	*R^2^*	*ΔR^2^*
1	(Constant)	.44	2.79					
	Caregiver SPSI-R Total Score	.72	.19	.39***	13.92***	.39	.14	.14
2	(Constant)	−5.30	3.55					
	Caregiver SPSI-R Total Score	.72	.19	.40***				
	Adolescent report CPI-SCI	1.31	.53	.25*	10.52***	.47	.22	.08

* *p* < .05.

** *p *< .01.

*** *p *< .001.

## 3. Results

Seventy-nine adolescent-caregiver dyads were included in analyses (Table 1). Adolescent participants were primarily male (55.7%), Caucasian (68.4%), with paraplegia (65.8%), and a mean age of 8.7 years at time of injury (SD = 5.6) and 15.8 years at time of interview (SD = 1.6). Caregiver participants were primarily female (87.3%), Caucasian (67.5%), married (53.8%), with at least some college (76.0%), and a mean age of 45.1 years at time of interview (SD = 6.5).

Table 2 presents descriptive statistics for adolescent problem-solving, caregiver problem-solving, and collaborative parent involvement. Both caregivers and adolescents reported moderate mean scores on the scales assessing positive and rational problem solving, with means ranging from 10.7–13.2. Caregivers reported low mean scores on negative problem-solving orientation (*M* = 4.5, SD = 3.9, Range = 0–19), impulsive/careless style (*M* = 4.5, SD = 4.1, Range = 0–20), and avoidant problem-solving style (*M* = 3.6, SD = 3.0, Range = 0–12). Adolescents reported low mean scores on negative problem-solving orientation (*M* = 4.7, SD = 4.3, Range = 0–18), as well as on impulsive/careless style (*M* = 6.2, SD = 3.5, Range = 0–15), and avoidant problem-solving style (*M* = 5.4, SD = 4.2, Range = 0–18). On average, adolescent participants reported high mean CPI-SCI scores, representing perceptions of care collaboration with their primary caregiver (*M *= 4.3, *SD *= .93, Range = 1–5).

Higher scores on caregiver positive or rational problem-solving subscales were weakly and positively correlated with higher scores on both adolescent positive and rational problem-solving subscales (*r *= .26 to.34, *p *< .05; Table 2). In addition, higher scores on caregiver negative and avoidant problem-solving subscales were significantly correlated with lower scores on adolescent rational problem-solving style (*r* = −.22 to -.33, *p *< .05). Further, higher scores on caregiver total problem-solving were significantly correlated with higher scores on adolescent PPO and RPS subscale scores (*r* = .33, *p* < .01; *r* = .40, *p* < .01, respectively). There was no significant correlation between caregiver NPO, ICS or AS subscale scores and adolescent PPO subscale scores. Greater adolescent perception of collaborative caregiver involvement in SCI care was significantly associated with higher scores on both adolescent positive and rational problem-solving orientation (*r *= .27, *p *< .05; *r *= .29, *p *< .05, respectively). Adolescent perceptions of CPI-SCI were not significantly associated with caregiver problem-solving subscales.

In general, significant differences across parent and adolescent report of problem-solving orientations were not identified. On average, adolescent report of positive problem-solving orientation was .61 points higher than caregiver report of positive problem-solving orientation (95% CI [−.58, 1.80]). Conversely, adolescent report of rational problem-solving orientation was .83 points lower than caregiver report of rational problem-solving orientation (95% CI [−2.06,.40]).

Multivariable linear regression analyses are shown in Tables 3, 4. Bivariate correlations across caregiver SPSI:R-S subscales justified use of the total caregiver SPSI:R-S score in regression models. Effective caregiver problem-solving and adolescent perceptions of collaborative parent involvement were each associated with positive problem-solving orientation in adolescents. Specifically, for each one unit increase in adolescent perceptions of collaborative parent involvement, there was a .27 unit increase in adolescent PPO scores when controlling for caregiver problem-solving (*p *< .05). The model with caregiver problem-solving as the sole predictor had an R^2^ value of .09 and the full adjusted model including both predictors had an R^2^ value of .16.

Effective caregiver problem-solving and adolescent perceptions of collaborative parent involvement were each associated with increased rational problem solving in adolescents. For each one unit increase in adolescent perceptions of collaborative parent involvement, there was a .25 unit increase in adolescent rational problem-solving scores when controlling for parent problem-solving (*p *< .05). The model with caregiver problem solving as the sole predictor has an R^2^ value of .14 and the full adjusted model including both predictors had an R^2^ value of .22.

## 4. Discussion

The current study is the first published account to examine predictors of constructive problem solving among adolescents with SCI. Results revealed that higher levels of adolescent positive problem-solving orientation and rational problem-solving style are both predicted by more effective problem solving by their caregivers, as well as adolescents perceiving more collaboration with caregivers. These findings provide a more nuanced understanding of the relationships between caregiver problem solving, collaborative involvement in SCI care, and constructive problem solving among adolescents with SCI, and have important implications for the development of effective problem-solving interventions for adolescents and caregivers. At present, there are few such interventions (15). Although this is a cross-sectional study, it is plausible that primary caregivers of adolescents with SCI who model effective problem solving, such as proactive engagement with a problem, can influence the development of those same skills in their adolescent. Pediatric rehabilitation programs could therefore consider the development of programming to help foster and reinforce these skills among caregivers.

This study is also the first to include an adapted measure of the perception of collaborative involvement of caregivers for adolescents with SCI. Collaboration could also entail flexibility such as modifying the caregiver's involvement in personal care assistance depending on the child's evolving care needs and development. In the current study, adolescents who perceived caregivers as their partners in meeting the care demands of living with SCI tended to have more constructive problem solving. These data may suggest that adolescents not only develop skills by observing their caregivers' approach to problem solving, but also through direct hands-on-learning as they work through care tasks together. These findings suggest the utility of an applied learning context in SCI care, where problem-solving strategies are modeled, shared, and developed.

### 4.1. Implications for clinical practice and intervention

Data suggest the potential promise of caregiver interventions aimed at fostering effective problem-solving skills and collaborative approaches to their child's care. This can be done during initial rehabilitation, and reinforced through long-term follow-up care and by all members of the adolescent's care team. For instance, staff can work to involve parents in treatment decisions, such as evaluating options and possible consequences. Staff can reinforce the importance of a growth mindset, including embracing and persisting through challenges, for parents by modeling persistence when trying new things and encouraging caregivers to approach problems as challenges to be solved. A growth mindset can help adolescents acquire more effective problem solving, and ideally improve quality of life. Guidelines for pediatric rehabilitation advocate for parent-involvement and family-centered care, though implementation has been limited (2, 20).

Raising a child with a disability heightens the dynamic nature of challenges faced by caregivers of typically developing children, in terms of balancing the desire to care for and protect their child with the need to foster independence. This may be particularly salient for youth who had already assumed some independence with regard to self-care behaviors at the time of injury. While there is no perfect “manual” for any parent, providing caregivers of youth with SCI with appropriate guidance specific to their child's age and level of impairment, as well as developmentally appropriate anticipatory guidance, can help caregivers prioritize competing demands, bolster rational problem solving, increase collaborative behaviors, and model effective problem solving for their youth and other family members.

Findings suggest a potential benefit of bolstering support for parents in development of problem-solving skills and constructive collaboration with their adolescent with SCI. Problem-solving interventions have demonstrated promise in other pediatric populations, including among caregivers of adolescents with chronic pain (21) and among caregivers of adults with SCI (14, 22, 19). Clinically, problem-solving interventions demonstrate feasibility and effectiveness in the context of pediatric chronic conditions and improve outcomes for both youth with chronic conditions and their parents, as well as for families of adults with disabilities (21, 22).

### 4.2. Strengths, limitations & suggestions for future research

This study examines predictors of constructive problem-solving among adolescents with SCI and describes the relationship between caregiver problem-solving approaches and constructive adolescent problem-solving. The results of the current study provide insight into factors that contribute to effective problem-solving for adolescents with SCI. This study includes the perspectives of both adolescents with SCI and their caregivers, which contributes to a more thorough understanding of the roles of problem-solving and perceptions of collaboration. Further, this study includes an adapted measure of the collaborative parent involvement survey specific to SCI. Not only can this adapted measure be used among other samples with SCI, but it also provides a model for how this measure can be adapted to other conditions.

The cross-sectional nature of the data limits the ability to evidence causation. Future research should employ longitudinal methods to further explore the temporal relations between caregiver problem-solving, collaborative parent involvement, and adolescent constructive problem-solving. Further, while all participants were part of the same health care system, they were recruited from three sites spanning a wide geographical area and varied socioeconomic status, which improves generalizability. Future research should seek to recruit a more diverse sample to enhance understanding of the role of contextual and social factors in family relationships, parent involvement, and problem solving skills.

Although adolescent perceptions of collaboration were related to adolescent problem-solving, caregiver perceptions of their own problem-solving strategies did not relate to collaborative involvement perceived by their adolescents. Future research should utilize diverse methods, such as observations and qualitative interviews, to assess parents' perceptions of collaboration to better understand the nature of collaboration, as well as barriers and facilitators to collaboration within the family context.

## 5. Conclusion

The current study highlights the important roles of caregiver problem-solving and collaboration around SCI care in constructive adolescent problem-solving. The results have important implications for interventions aimed at fostering effective problem-solving skills among caregivers and implementing collaborative approaches to SCI care.

## Data Availability

The datasets presented in this article are not readily available because; Shriners Children's does not allow for sharing of the dataset. Requests to access the datasets should be directed to; kzebracki@shrinenet.org.
